# ACMQ Student, Resident, and Fellow Section (SRF) COVID-19 Resident and Fellow Survey

**DOI:** 10.1177/1062860620926271

**Published:** 2020-05-16

**Authors:** Michael Jin, Christopher Jackson, Assim M AlAbdulKader, Kevin Hummel, Jeffrey Kott, Oliver Nguyen, Sohayla Rostami, Sun Young Kim

**Affiliations:** 1 Radiology Resident, Stony Brook University Hospital NY; 2 Internal Medicine Physician, University of Tennessee, Memphis TN; 3 Family & Preventive Medicine Physician and Geriatrics Fellow at CWRU/MetroHealth Medical Center OH; 4 Pediatric Critical Care Fellow, University of Utah UT; 5 Internal Medicine Chief Resident, Stony Brook University Hospital NY; 6 University of Alabama at Birmingham AL; 7 General Surgery Resident, St John’s Episcopal Hospital, Queens NY; 8 Division of Hematology/Oncology/Stem Cell Transplant, Ann and Robert H. Lurie Children’s Hospital IL

The current COVID-19 outbreak has become an enormous global public health concern for patients and their health care providers, especially for physicians-in-training who are delivering care on the frontlines of this pandemic. While COVID-19 continues its grip on nearly all aspects of our daily living, questions remain. How long this crisis will last? When are we going to go back to our normal lives? As leaders of the ACMQ Student/Resident/Fellow Section (SRF), we feel that it is critically important to evaluate the opinions of Resident and Fellow physicians in training across the United States regarding a number of important issues related to proper infection control, organizational readiness for combating and containing COVID-19, and, most importantly, clinician wellness. To do this, we created an online anonymous survey in late March 2020 and disseminated a hot link to it widely to many of our colleagues across the United States. As SRF Leaders, we strongly believe it is extremely important to highlight and emphasize the opinions and points of view of physicians-in-training, who are currently on the frontlines of the US health system every day doing everything in their power to ensure the delivery of high-quality, safe care in the tireless quest to end the COVID-19 pandemic. While we are still collecting new data on a daily basis, we intend to disseminate the results of this survey once we have closed the survey in the next several weeks. Some of the questions and topics that we are addressing through this survey include the following:

1. *Organizational Awareness and Readiness*: What was your institution’s level of concern related to COVID-19 aa the first patient was diagnosed in January 2020 and how did this change in February and March as the pandemic unfolded.
2. Which source(s)/organizations provide residents and fellows with the most reliable and timely information on COVID-19?
3. What are the top 3 concerns currently regarding clinical work as a resident/fellow related to COVID-19?
4. What do residents and fellows believe about the reported number of patients COVID-19 from websites, social media, or the news compared with the actual number of patients (including those not tested or diagnosed)?
5. Which interventions were initially most important when COVID-19 infections were first reported in the United States and then later as the pandemic expanded its global course in March and April 2020?
6. Which intervention have the most promise in the next 3 to 6 months for dramatically reducing COVID-19 infections?
7. If COVID-19 were to be successfully contained in the next few weeks, estimate how long it would take for your institution to get back to the regular business as usual?
8. How are you feeling today about your clinical work in the face of COVID-19?

Some of our initial observations from the findings of this survey include the following:

- Not surprisingly, the most common feeling among frontline residents and fellows in clinical training in the face of COVID-19 is anxiety, as all providers are being placed in situations with a level of responsibility that they may have never truly anticipated having. Whether it be witnessing the death of someone far too young and having to break the news to family over the phone, or providing support to a scared individual who is unable to be with any family or friends around due to institutional policies restricting visitors. These anxious feelings may also be compounded by the scary thought that in some parts of the United States, health care providers (including residents and fellows) could be contributing to the spread of the virus.
- During clinical work, residents are currently most worried about contracting COVID-19 at work or spreading it to family, friends, colleagues, and patients. This is a significant risk especially given the early challenges of lack of personal protective equipment (PPE) and patient care equipment such as ventilators in hard-hit areas. Many respondents now seem less anxious and concerned about not being able to conduct patient care or having processes to care for patients infected with COVID-19, suggesting that they feel that their own hospitals now have adequate levels of PPE and effective evidence-based protocols in place for isolating and caring for these patients.
- Over half of respondents to date (54%) indicate expectations that the reported number of COVID-19 patients in public media is likely to be 10 times higher than the number currently reported across public and scientific media channels. While this reflects the evolving use of more widespread population-based testing, trainees on the frontline may be worried about a further surge of COVID-19 exposure that could have a profound impact on their training and educational experiences.
- Many more respondents feel supported than unsupported in their daily work. While a few are feeling angry and depressed, others are optimistic and hopeful. Also, 43% of trainees currently think it will take 1 to 3 months for their institutions to get back to the regular business as usual, while 31% believe it could take 3 to 6 months.

If you are currently a resident or fellow in training, we strongly encourage you today to participate in this survey! Please use this link https://acmq.org/news/news.asp?id=502129&hhSearchTerms=%22covid%22 to access the ACMQ SRF COVID-19 Survey or contact SRF Chair Dr. Mike Jin at michael.jin@stonybrookmedicine.edu for assistance.

If you are currently not a member of ACMQ SRF, please be sure to visit the ACMQ SRF Webpage at https://acmq.org/page/StudentResident to join ACMQ and stay current with the latest information on SRF activities.

And Thank You All for your courageous service and commitment to high-quality, excellent patient care!

